# Carbon dynamics and GHG implications of increasing wood construction: long-term scenarios for residential buildings in Austria

**DOI:** 10.1080/17583004.2018.1469948

**Published:** 2018-05-29

**Authors:** Gerald Kalt

**Affiliations:** a Institute of Social Ecology, University of Natural Resources and Life Sciences, Vienna, Austria; b Austrian Energy Agency, Vienna, Austria

**Keywords:** GHG accounting, carbon savings, material substitution, material stocks, wood construction, dynamic stock model

## Abstract

Wooden construction elements often exhibit lower life cycle greenhouse gas (GHG) emissions than conventional counterparts (‘material substitution effect’). Moreover, the building stock represents a carbon (C) sink if timber inflows (construction) surpass outflows (demolition) (‘C-stock effect’).

A dynamic stock model incorporating these effects is applied to quantify potential climate benefits of wood construction in Austria's residential building sector. If present trends are maintained, culminating in a wood construction share (WCS) of 50% during 2050-2100, building shells could contain three times as much C in 2100 as today. Annual timber demand for residential construction could double, but would remain well below Austria's current net exports. Compared to a baseline scenario with constant WCS (22%), cumulated GHG savings from material substitution until 2050 are estimated 2 to 4.2 Tg CO_2_-equivalent – clearly less than savings from C-stock expansion (9.2 Tg). Savings from both effects would double in a highly ambitious scenario (WCS=80% during 2050-2100).

The applied ’Stock Change Approach’ is consistent with IPCC Guidelines, but the above-mentioned savings from C-stock changes would not materialize under the current default GHG inventory accounting approach. Moreover, savings from C-stock effects must eventually be weighed against forest C-stock changes, as growing domestic demand might stimulate wood harvesting.

## 1 Background and motivation

### 1.1 Environmental benefits of wood-based products and buildings

There is broad scientific evidence for environmental benefits of wood-based products compared to conventional counterparts consisting of ‘non-wood’ (metallic, synthetic or mineral) materials [1]. In a meta-study focusing on greenhouse gases (GHG), Sathre and O'Connor [2] conclude that for each ton of carbon in wood products substituted in place of non-wood products, savings are typically in the range of 1.8 and 5.5 t CO_2_-eq. Werner and Richter [3] conducted an extensive literature review of comparative life-cycle assessments (LCA) and found that, with regard to most impact categories, wood products ‘tend to have [a] favorable environmental profile’. This is confirmed by, for example, Bergman *et al*. [4], who state that ‘wood products have many environmental advantages over non-wood alternatives’ and found ‘notable carbon emissions savings when wood products are used in constructing buildings’. Many more studies investigating functionally equivalent building types and construction elements from a life-cycle perspective have demonstrated that wood-based construction usually requires less energy and generates less GHG emission than buildings mainly constructed from other materials [5–9,101]. Suter *et al*. [10] analyzed the complete value chain of all wooden goods produced or consumed in Switzerland; they concluded that replacement of energy-intensive building materials is one of the wood applications with the highest GHG savings. Braun *et al*. [11] conducted a scenario-based assessment for the Austrian wood chain and found that ‘material use of products from domestic timber sources has the highest climate mitigation efficiency’, provided that forests are managed sustainably. However, this study followed a supply-based approach and did not focus on possible future developments in wood construction or any other demand sector.

The beneficial properties of wood products, construction elements and buildings are the result of two separate mechanisms. The first is a usually lower ‘carbon footprint’, meaning the quantity of GHG emissions during a product's manufacturing and, in some cases, end-use and disposal [4,12]. This aspect is usually termed ‘product/material substitution’ [cf. 2,13,14]. And, second, is the ‘carbon stock/pool/storage effect’; it refers to the fact that carbon remains fixed in wood products throughout their service life. This is especially relevant for wood-based construction, as typical service lives of buildings amount to many decades [15–17].

### 1.2 Wood construction in Austria

The share of wood-based buildings in Austria has increased continuously during the last two decades: According to Teischinger *et al*. [18] 9% of the total residential building (RB) volume erected in Austria 1998 was attributable to wood buildings. In 2013 the wood construction share (WCS) had increased to 21%.

The market potential of wood buildings appears to be far from fully exploited: So far, the trend to wood construction was strongest in the market segment of single-family houses in rural areas. In urban areas with higher shares of multi-family buildings, wood construction is less relevant, as data for the city of Vienna indicate. Nevertheless, the number of wood construction projects in Vienna has increased by almost 50% from 2003 to 2013 [18]. This development, and demonstration projects for tall wood buildings (e.g. the ‘HoHo Vienna’, a 24-floor lighthouse project in the urban expansion area Aspern [cf. 102]) are indications that the trend to wood construction might continue throughout the next few decades. Increased efforts to reduce GHG emissions, which will be necessary for achieving the ‘Paris Agreement’ [103, cf. 19,20], and to establish a ‘circular’ or ‘bio-economy’ until 2050 [21,22] might even accelerate this trend.

### 1.3 Previous studies on a national scale

Although there is a plethora of comparative LCA literature focusing on the carbon benefits of wood construction, the potential relevance for climate mitigation on a national scale has hardly been explored yet. Two studies on this issue, for the cases of Germany [15] and Switzerland [23], investigated the GHG implications of (relatively moderate) increases in wood construction. Assuming a maximum WCS of 55% for single- and two-family houses and 15% for multi-family houses throughout 2016 to 2030, the analysis for Germany indicates cumulative GHG savings of up to 43 Mt CO_2_-eq. during the considered time frame (sum of carbon stock and substitution effects). The corresponding average annual savings are equivalent to about 0.3% of Germany's total annual GHG emissions in 2014 to 2016 [24]. The study for Switzerland puts more emphasis on long-term dynamics by considering the time frame until 2130. The authors of this study found that ‘an increased use of wood in the building sector is a valid and valuable option for the mitigation of GHG emissions … on a mid- to long-term basis’. Interestingly, they also concluded that the carbon stock effect is of minor importance compared to substitution effects [23].

## 2 Research questions

This work seeks to quantify the potential long-term climate benefits resulting from a further increase in wood construction in the Austrian RB sector. Savings from material substitution as well as from carbon stock changes are considered. As in Kalcher *et al*. [16], the scope of the assessment includes all wood contained in the shells of RBs. Interior and exterior finishing, furniture, doors, etc. as well as non-residential buildings are not considered.

Interim results include consistent long-term scenarios for timber and carbon stocks as well as for construction wood demand and waste wood quantities from building demolition. These results might be valuable inputs for the research fields of material flow and stock analysis and social metabolism [see e.g. 25,26].

Due to considerable uncertainties with regard to numerous factors involved, the objective is to explore possible ranges and provide insight into implications of different long-term developments. Following the principles of scenario analysis, contrasting alternative futures are derived, some of which might appear quite unlikely from today's point of view.

Forest carbon stocks and GHG savings from fuel substitution (i.e. fossil fuel replacement with demolition wood) are not within the scope of the model. To what extent these system boundaries might limit the validity of the results is discussed in the final section.

## 3 Methodology and data

### 3.1 Methodological approach

The methodological approach consists of the following steps:

- **Data research and selection of primary data:** In order to model the building stock, comprehensive data on the current structure of the RB sector are required. A study by Kalcher *et al*. [16] was identified as an excellent reference in terms of data as well as methodologically. Further data sources are described below and summarized in the Appendix (Table A1);
- **Definition of scenario parameters and scenario spectrum:** Scenarios vary in their projections for average floor space as well as for the WCS (which is translated into an average ‘timber intensity’). A single projection for population development was used for all scenarios. Uncertainties regarding GHG savings of wood construction in comparison to conventional styles (i.e. regarding the ‘substitution factor’) are addressed within sensitivity analyses;
- **Modelling building and timber stocks:** Based on a methodological framework developed by D. B. Müller [17], annual stock inflows and outflows and the resulting total timber stocks are calculated for the time frame 2010 to 2100;
- By converting timber stock developments into equivalent values of carbon and CO_2_, **climate benefits resulting from carbon stock increases** are quantified;
- **GHG reductions resulting from material substitution** are calculated on the basis of specific GHG savings per m^2^ of floor space derived from LCA data in the literature;
- The **total GHG savings**, calculated as the sum of the two components, is analyzed on an annual and cumulated basis for each scenario.

### 3.2 Building and timber stock dynamics model

A slightly modified version of the model described by D.B. Müller [17] is applied. Calculations are performed on an annual basis. This is why discrete-time notation is used instead of Müller's continuous-time notation.

The model involves population development (‘p’), floor space (‘s’) and wood being used as construction material (i.e. timber, ‘m’). The according time-dependent state variables K^(p)^(t), K^(s)^(t) and K^(m)^(t) represent the total population, the existing floor space and the total stock of timber in building shells, respectively, in the year ‘t’. Furthermore, the input (I^(s)^(t), I^(m)^(t)) and output flows (O^(s)^(t), O^(m)^(t)) of floor space and timber are considered. Outflows are due to building demolition and inflows due to construction. The relationships between population and floor space is characterized by the determinant ‘floor space per capita’, ($K_{c}^{\left(s\right)}(t)$), and the relationship between floor space inflows/outflows and timber flows by the average ‘timber intensity’ ($M_{s}^{\left(m\right)}(t)$) in the year of construction. The scenarios are based on different assumptions about future developments of these determinants.

Floor space development is linked to population development:

(1) $$K^{\left(s\right)}\;\left(t\right)=K^{\left(p\right)}\;\left(t\right){\cdot K}_{c}^{\left(p\right)}\left(t\right)$$

Hence, inflows of floor space (i.e. building construction) must eventually compensate for increasing demand due to population growth and changing average floor space per capita, as well as age-related stock outflows (building demolition). The balance equations, describing the relationship between stock changes, stock inflows and outflows, are considered for floor space as well as timber:

(2) $$K^{\left(s\right)}\;\left(t\right)=K^{\left(s\right)}\;\left(t-1\right)+I^{\left(s\right)}\left(t\right)-O^{\left(s\right)}\left(t\right)$$

(3) $$K^{\left(m\right)}\;\left(t\right)=K^{\left(m\right)}\;\left(t-1\right)+I^{\left(m\right)}\left(t\right)-O^{\left(m\right)}\left(t\right)$$

Outflows of floor space are determined by inflows in the past and an assumed lifetime distribution $\left(L\left(t,t'\right)\right)$:

(4) $$O^{\left(s\right)}\;\left(t\right)=\sum_{{t^{'}=t}_{0}}^{t-1}L\left(t,t'\right)\cdot I^{\left(s\right)}\left(t'\right)\;$$

t_0_ denotes the construction year of the oldest existing buildings. The lifetime of buildings is assumed to be normally distributed with a standard deviation σ of 20 years (adopted from the case study in [17]; see also section 1.4 of the supporting information to Krausmann *et al*. [26]). The expected/mean value of building lifetimes τ is assumed to be 120 years for buildings constructed before 1945, and 80 years for the rest (the ‘standard scenario’ according to Kalcher *et al*. [16]):

(5) $$L\;\left(t,t'\right)=\frac{1}{\sqrt{2\pi \sigma^{2}}}\;e^{\frac{-{\left(t-t^{'}-\tau \right)}^{2}}{2\sigma^{2}}}$$

Inflows and outflows of timber are directly linked to inflows and outflows of floor space through timber intensity in the respective construction year:

(6) $$I^{\left(m\right)}\;\left(t\right)=I^{\left(s\right)}\;\left(t\right)\cdot M_{s}^{\left(m\right)}\left(t\right)$$

(7) $$O^{\left(m\right)}\;\left(t\right)=\sum_{{t^{'}=t}_{0}}^{t-1}L\left(t,t^{'}\right)\cdot I^{\left(s\right)}\left(t^{'}\right){\cdot M}_{s}^{\left(m\right)}\left(t^{'}\right)\;$$

$I^{\left(m\right)}\left(t\right)$ represents the consumption of construction wood and $O^{\left(m\right)}\left(t\right)$ the amount of waste wood from building demolition in the year t.

### 3.3 Data sources and scenario parameters

**Figure 1.**
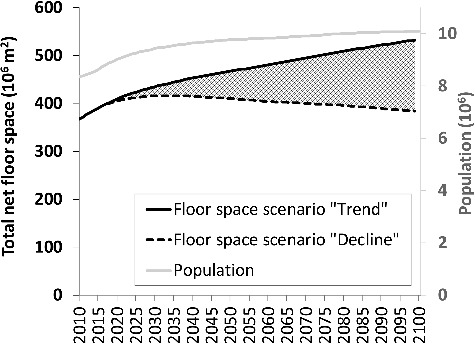
Population forecast according to Statistik Austria [104]) and alternative developments in total net floor space in residential buildings in Austria (scenarios ‘trend’ and ‘decline’).

Population in Austria is assumed to develop according to the most recent forecast from the national statistical authority [104] (Figure 1). Floor space per person has shown a significant increase in recent decades [105]. Two diverging projections are assumed in the scenarios: a further increase (‘trend scenario’) from 44.6 m^2^ per person in 2015 to 47.7 in 2050 and 52.7 in 2100 (adopted from A. Müller *et al*. [27] and the ‘moderate increase’ scenario according to Kalcher *et al*. [16]); or a contrasting development showing a trend reversal after 2020 with a decline to the level of 2001 until 2100 (adopted from Kalcher *et al*. [16]: ‘moderate decline’ scenario). The resulting developments in total floor space demand are also shown in Figure 1. The dashed area between the two floor space projections indicates the range represented in scenario calculations.

Data on timber intensities until 2010 and on age distribution of the building stock in 2010 are available from Kalcher *et al*. [16]. Aggregated average timber intensities in 2011 to 2015 have been estimated using empirical data on WCS according to Teischinger *et al*. [18] and benchmark values for standard building types derived from the national construction element database ‘Baubook’ [106]. The results are in agreement with timber intensities in the period 2001 to 2010 according to Kalcher *et al*. [16].

**Figure 2.**
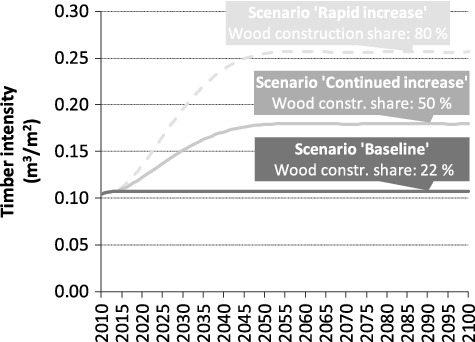
Scenarios for the development of average timber intensities in residential building construction.

Three different projections for the wood construction share WCS (and corresponding average timber intensities) after 2015 are assumed (Figure 2): first is a ‘baseline’ scenario with a constant share of 22% throughout the whole time frame. The corresponding average timber intensity is 0.108 m^3^ per m^2^ net floor space. Second is a scenario with a continued increase at approximately the same rate observed in recent years and saturation at WCS = 50% around 2050 (‘continued increase scenario’). And the third is a projection with an accelerated trend to wood construction, resulting in a WCS of 80% in 2050. The shapes of the latter two projections shown in Figure 2 have been derived by applying polynomial curve fitting to historical data points and the respective saturation value for the time frame 2050 to 2100. The resulting S-shaped curves are considered appropriate approximations for market diffusion dynamics.

Since timber is used not only in wood construction but also in conventional building shells, a doubling of the WCS does not correspond to a doubling of the timber intensity.

### 3.4 GHG savings

With regard to wood conversion factors (average carbon contents, specific weight), default conversion parameters according to IPCC guidelines are used [107].

Material substitution factors are based on a recent study by Hafner and Schäfer [5]. It provides life-cycle emissions for functionally equivalent buildings suitable for deriving representative per-m^2^ GHG savings. Despite the wealth of comparative LCA studies on the topic of wood construction [cf. 2], the results from Hafner and Schäfer [5] are the only ideal data for the current study. This is for three reasons: they are up-to-date; they refer to buildings constructed in Germany and Austria, and hence they can be considered representative for Austrian building styles; and the study focusses on GHG emissions from the ‘production’ and ‘end-of-life’ stages (modules ‘A’ and ‘C’ in the LCA scheme according to the norm DIN EN 15978:2012), which is consistent with the system boundaries of the present approach.

For single- and two-family buildings, GHG savings according to Hafner and Schäfer [5] range from 77 to 207 kg CO_2_-eq./m^2^, corresponding to relative savings between 35 and 56%. The range for multi-family buildings is even wider: 18 to 178 kg CO_2_-eq./m^2^ (corresponding to 9–48% relative savings). Based on the median values and a weighted average of single-/two-family and multi-family houses, the following rounded default value for the base year 2015 was derived: s^(ms)^ (2015) = 100 kg CO_2_-eq. per m^2^ gross floor space.

As these savings refer to current production processes, energy mixes, etc., it is considered inappropriate to assume that they are valid for the entire time frame. With regard to efforts to reduce GHG emissions throughout all sectors and industrial processes [28–30], it is more likely that specific GHG savings from material substitution will decline, as production processes become more efficient, carbon-intensive energy sources are phased out, etc.

Systematic approaches for deriving dynamic life-cycle coefficients have only recently been developed in the context of energy systems [31,32]. Studies for specific building types are still lacking. Hence, the simplistic assumption is made that the construction sector and all processes and activities relevant for building construction are ‘decarbonized’ at the same pace as the EU intends for the entire economy: minus 80% until 2050 (the minimum requirement according to [29]). The assumed time-dependent (‘dynamic’) substitution factor is described by the following equation:

(8) $$s^{\left(ms\right)}\;\left(t\right)=s^{\left(ms\right)}\;\left(2015\right)\cdot \left(1-80\text{\%}\cdot \frac{t-2015}{2050-2015}\right)$$

A sensitivity analysis is dedicated to this assumption.

Annual GHG savings from material substitution $\left(S^{\left(ms\right)}\left(t\right)\right)$ are calculated from floor space additions in wood buildings in each year:

(9) $$S^{\left(ms\right)}\;\left(t\right)=\frac{I^{\left(s\right)}\left(t\right)\cdot s^{\left(ms\right)}\left(t\right)\cdot p^{\left(wc\right)}\left(t\right)}{\beta }\;$$

β is a constant for converting net floor space (used in the context of floor space demand) to gross floor space. By default, β is assumed to be 0.7 [cf. 33,34].

GHG savings from carbon stock increases $\left(S^{\left(cs\right)}\left(t\right)\right)$ are calculated from material stock changes:

(10) $$S^{\left(cs\right)}\;\left(t\right)=\left(K^{\left(m\right)}\left(t\right)-K^{\left(m\right)}\left(t-1\right)\right)\;\cdot \gamma$$

with material stocks being measured in m^3^ of wood and γ being a constant conversion factor of 0.825 t CO_2_-eq./m^3^ (based on [107]). Total GHG savings are the sum of the two components S^(ms)^ (t) and S^(cs)^ (t).

### 3.5 Context to ‘HWP accounting’

Regarding carbon stock accounting, the methodology described above is basically consistent with IPCC Guidelines on ‘HWP (harvested wood product) accounting’, as these guidelines define some general rules and good practice guidance but also leave methodological options open. The method applied here can be characterized as a ‘stock change approach’ (SCA) [cf. 35,36] with normally distributed ‘decay’. While first-order exponential decay is the default Tier 2 approach, assuming other distribution functions is expressly permitted under Tier 3 [107].

Contrary to the approach used in this study, the ‘production approach’ (PA) was applied and ‘first-order decay’ assumed for HWP calculations in the latest Austrian GHG inventory report [37]. The PA is based on production statistics for sawnwood, wood-based panels and paper, and attributes the corresponding amount of carbon to the producer country [cf. 38]. The fact that exported wood products are transferred to another country (i.e. they actually contribute to the importer country's carbon stock change) is disregarded. In other words, the PA is ignorant of where wood products are actually utilized. By considering production instead of consumption quantities, it disregards actual carbon stock changes within the country under consideration [cf. 35]. For the research question at hand it is therefore reasonable to deviate from the current default approach and apply a stock change approach, which captures actual carbon stock changes within national boundaries.

## 4 Results

Results are shown for time frames starting with 2015. While results referring to building stock developments and wood quantities are shown until 2100, calculations regarding GHG savings refer to the time frame until 2050, in order to focus on the time horizon usually considered in connection with climate policy targets.

### 4.1 Construction and demolition wood

**Figure 3.**
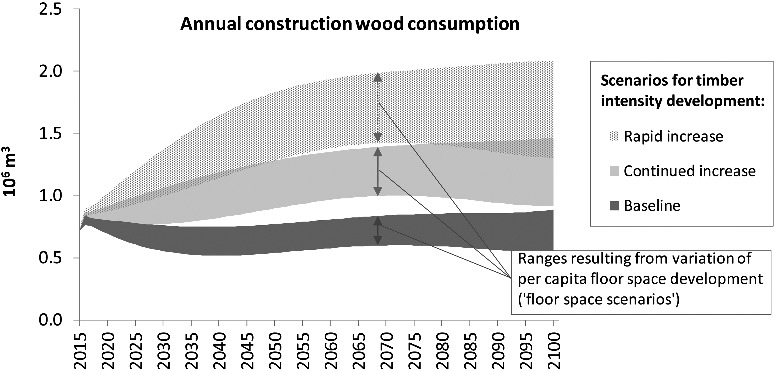
Annual construction wood consumption in the scenarios.

Figure 3 shows the annual consumption of construction wood in the main scenarios. The results for the three timber-intensity scenarios are shown as (partly overlapping) areas reflecting the ranges due to variation of per-capita floor space (projections shown in Figure 1). As the figure shows, the ‘baseline’ scenario with increasing per-capita floor space (upper boundary of the dark gray area) is characterized by a relatively constant annual timber demand between 0.7 and 0.9 million m^3^ (Mm^3^). In the case of a ‘rapid increase’ in wood construction the demand could more than double until the middle of the century and account for more than 2 Mm^3^ in 2100, if per-capita floor space continues to increase.

**Figure 4.**
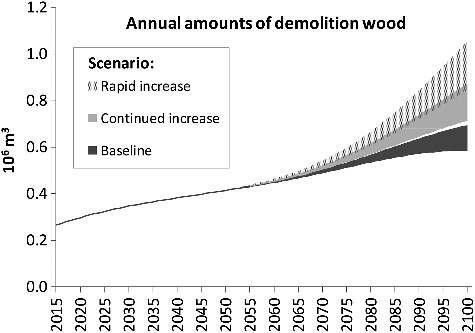
Annual amounts of waste wood from residential building demolition in the scenarios.

With regard to annual amounts of waste wood from building demolition (Figure 4), the scenarios are almost identical until 2050, as the development is almost exclusively determined by the historical building stock during the first half of the century. In the second half, however, the diverging trends in building construction start to take effect: In the last few years of the century, a range from 0.6 to more than 1 Mm^3^/a is covered by the scenarios. Hence, even in the ‘baseline’ scenario with low floor space demand, annual amounts of demolition wood increase by more than 100%.

**Figure 5.**
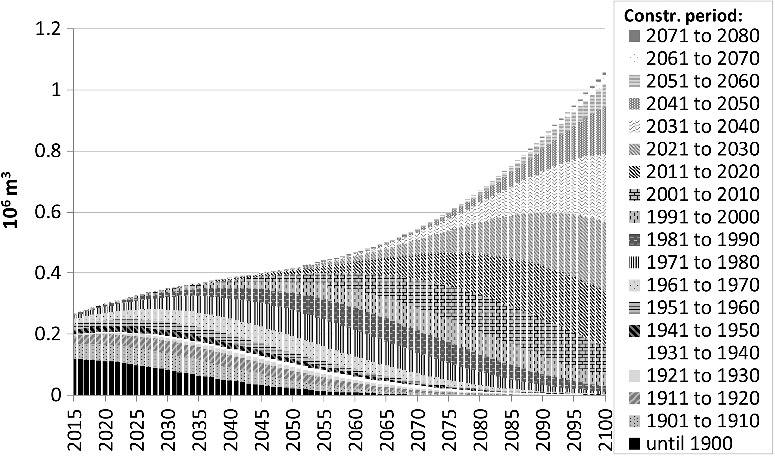
Annual amounts of waste wood in an exemplary scenario, broken down by construction period.

Figure 5 shows the demolition wood quantities for one exemplary scenario (‘rapid increase’; floor space scenario ‘trend’) broken down by 10-year-construction periods. It illustrates that – according to the model – current amounts of demolition wood are mainly determined by construction activity and practices in the 19^th^ and early 20^th^ centuries. Timber intensity was relatively high in this time frame. Thereafter, it decreased by more than 50%. A relatively small share of the current stock originates from the period 1919 to 1944 [16], which explains the low relevance of the corresponding components in Figure 5. In the second half of the 21^st^ century, rapidly increasing timber intensities in this scenario start to take effect, resulting in a steep increase.

### 4.2 Carbon and timber stock developments

**Figure 6.**
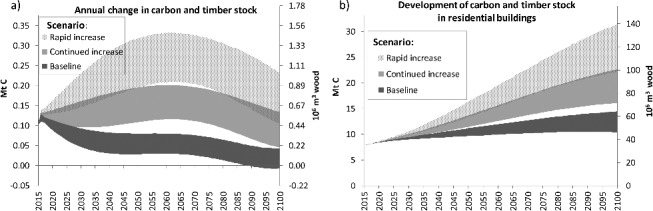
Development of annual wood and carbon stock changes (a) and total carbon and timber stocks (b) in Austrian residential buildings in the scenarios.

Figure 6(a) illustrates the annual change in carbon and timber stock in RB, and Figure 6(b) shows the resulting development in carbon and timber stocks until 2100. The ‘baseline’ scenario, with a constant WCS of 22%, shows an increase from 7.8 million tons of carbon (Mt C) in 2015 to 10.4 Mt in the lowest and 14.4 Mt in the highest case until 2100. With a ‘continued increase’ in timber intensity and saturation at WCS  = 50%, the carbon stock would increase about two- to threefold until 2100. In the case of a ‘rapid increase’ in wood construction, the carbon stock in RB could amount to more than 31 Mt C at the end of the century. This corresponds to 140 Mm^3^ of wood, a fourfold increase compared to 2015, and more than a tenth of the standing wood (stemwood with bark) in Austrian forests [39].

### 4.3 GHG savings from increase in wood construction

**Figure 7.**
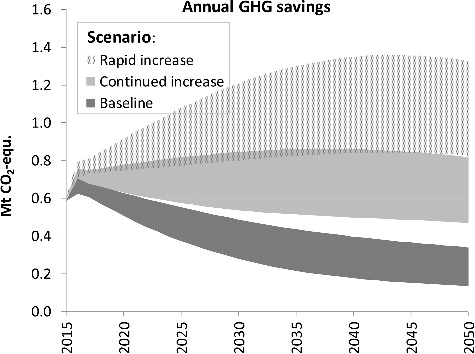
Annual GHG savings from wood construction in the scenarios.

The total annual GHG savings, resulting from material substitution as well as carbon stock effects, are shown in Figure 7. The downward trend in the ‘baseline’ scenario is due to decreasing per-m^2^ savings from material substitution. In the ‘continued increase’ scenario the total savings remain relatively constant, as decreasing savings from material substitution are more or less counterbalanced by rising savings from carbon stock developments. And in the case of a ‘rapid increase’ in wood construction, the scenarios span a range from almost constant savings (of about 0.8 million tons (i.e. Tg) CO_2_-eq./a) to an upswing to 1.35 Mt CO_2_-eq./a.

**Figure 8.**
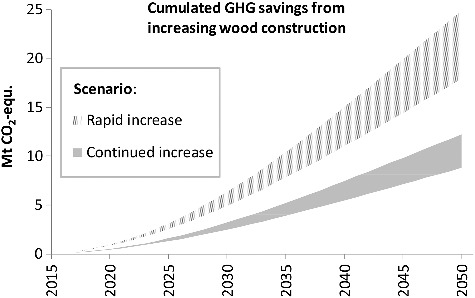
Cumulated GHG savings in the scenarios ‘rapid/continued increase’ relative to the baseline scenario.

Figure 8 provides the answer to the core questions of this work. It shows the total cumulated GHG savings in the ‘rapid’ and ‘continued increase’ scenarios, relative to the ‘baseline’ scenario. During the first couple of years the climate mitigation effect is rather limited. Even in the ‘rapid increase’ scenario, with a relatively steep WCS increase, it takes some time for the cumulated savings to reach substantial levels. But in the longer term, annual savings could amount to up to 1 Mt CO_2_-eq./a in the ‘rapid increase’ scenario and close to 0.5 Mt CO_2_-eq./a in the ‘continued increase’ scenario. The corresponding cumulated savings in 2050 are in the range of 18 and 25 Mt CO_2_-eq. and 8.8 and 12.3 Mt CO_2_-eq., respectively. More than 60% of the cumulated savings are due to the carbon stock effect.

### 4.4 Sensitivity: substitution factor

**Figure 9.**
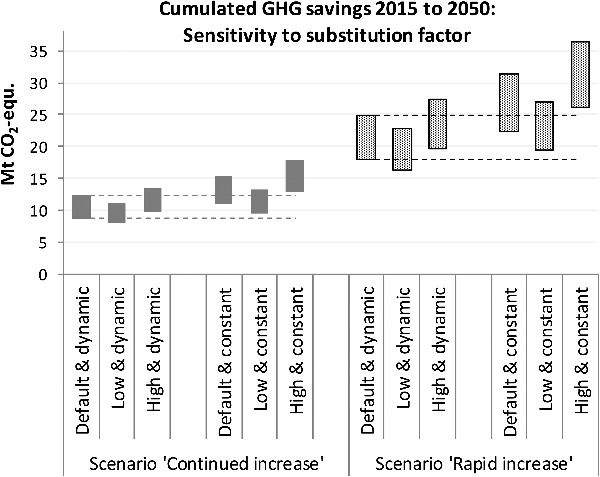
Results of sensitivity analyses regarding GHG savings from wood construction: Ranges of cumulated savings during 2015 to 2050 in the standard scenarios (‘default and dynamic’) compared to alternative cases.

Comparative LCA studies indicate that specific GHG savings of wood construction compared to conventional building types vary widely; depending on building types and designs, relative savings range from less than 10% to more than 50% [5]. The default value assumed in the above calculations (100 kg CO_2_-eq./m^2^ gross floor space) is considered a good estimate for typical savings from material substitution. Nevertheless, it is uncertain, and it could be argued that the assumed linear decline of the substitution factor (by about 2.3% p.a.; see Equation 8) is quite speculative. The assumed average ratio of net floor space to gross external area β in Equation 9 adds to this uncertainty. In the sensitivity analysis presented in Figure 9, the following alternative cases are investigated: A ‘high’ and a ‘low’ assumption for the initial (2015) substitution factor, and ‘constant’ factors instead of a linear decline (denoted as ‘dynamic’). ‘High’ refers to a substitution factor of 0.13 t CO_2_-eq./m^2^ and an average ratio of net to gross floor pace of 0.65, while ‘low’ refers to 0.07 t CO_2_-eq./m^2^ and 0.75 m^2^ net per m^2^ gross. In Figure 9, the cumulated GHG savings during 2015 to 2050 based on the standard (‘default and dynamic’) and alternative cases are compared for both timber intensity scenarios (‘continued’ and ‘rapid increase’).

It is concluded that a variation of the initial value within reasonable ranges (‘high/low and dynamic’) leads to quite moderate deviations from the default results (about ± 10%). The reasons are that that total savings are largely determined by timber stock increases, while the relevance of the substitution effect gradually declines. In later decades, when the deviation from the ‘baseline’ scenario is highest, the savings from stock increases clearly dominate over the substitution effect. This is not the case if constant substitution factors are assumed. Under this assumption the total savings are up to 36% higher than in the respective scenarios with ‘dynamic’ substitution factors.

However, it is considered highly unlikely that substitution factors will remain constant until 2050. In order to meet climate policy targets, it is necessary to reduce GHG emissions throughout all sectors and industrial processes, leading to declining life-cycle emissions for all kinds of products, construction elements and buildings. Progress in decarbonization might differ considerably between sectors and product groups, and the assumption of an 80% decline until 2050 is of course simplistic. But it is obvious that assuming constant substitution factors results in a significant overestimation of actually achievable GHG savings.

The numerical values of cumulated GHG savings in all scenarios are provided in Table A2 in the Appendix.

## 5 Discussion and conclusions

Estimates of the potential long-term contribution of wood construction to climate mitigation are difficult, as they depend on numerous uncertain factors. Still, the presented scenarios provide valuable insight and lead to several – quite robust – conclusions.

### 5.1 Assumptions and implications

As a result of historic developments, the annual amounts of demolition wood (available for energy generation or material uses) will increase significantly during the 21^st^ century. The ‘baseline’ results are in good agreement with the standard scenario by Kalcher *et al*. [16], although they are based on different probability distributions of building lifetimes: If the WCS remains constant, annual demolition wood quantities increase by about 100% until 2100, whereas the demand for construction wood remains relatively stable. The timber (and carbon) stock in RB shells, currently estimated at about 8 Mt C, increases by up to 85%.

In the case of a continued (or even accelerated) trend to wood construction, and a sustained share of 50% (80%) throughout the second half of the century, more than 20 Mt (30 Mt) of carbon could be stored in RB shells by 2100. Considering the growth rates during the last two decades [18] and policy targets to establish a circular/bio-economy [21,22], a WCS of 50% by 2050 appears possible. With its strong, currently export-oriented wood industry [40,41], Austria has ideal conditions; and in comparison to, for example, Scandinavian countries, the WCS is currently still quite low. In Sweden 90% of all single-family houses are constructed of wood [42], and in Finland the share of wood-framed buildings in the market segment of detached houses is 80% [43]. Efforts to increase the WCS in multi-storey buildings are being made and are already showing an effect in these and other countries [42, 43], demonstrating the viability and providing international showcase projects. Provided that long-term policy targets related to GHG mitigation and sustainable development are pursued consistently and with strong policy instruments in place, a development similar to the ‘rapid increase’ scenario might even become possible.

To put the additional construction wood demand in the ambitious scenarios into perspective with current wood streams, it is reasonable to compare them with Austria's net exports of sawnwood and panelboard. In total, they accounted for about 6 million m^3^ per year [108], while the additional annual demand in the second half of the 21^st^ century is less than 0.5 million m^3^ in the ‘continued increase’ scenario and about 1 million m^3^ in the ‘rapid increase’ scenario. Hence, at its current production output, the Austrian wood industry could quite easily supply the required amounts.

### 5.2 Methodological aspects and system boundaries

Current IPCC guidelines provide a framework for accounting carbon stock in wood products (‘HWP accounting’). According to IPCC [107], different approaches may be applied for calculating carbon stock changes in HWP and corresponding GHG emissions or removals. The results from different approaches can vary widely, as studies have shown [38,44,45]. Variations are particularly large for countries with high import or export ratios, such as Austria.

For this study, the ‘stock change approach’ was selected because it considers actual inflows and outflows to and from the domestic HWP stock. The ‘production approach’, which is applied in the Austrian GHG inventory [37], is ignorant of where stock changes actually take place. It is therefore not suitable for investigating the research question at hand. If this accounting approach is maintained, carbon savings calculated here would not materialize in the national GHG inventory [cf. 13].

Another important methodological aspect is related to forest carbon stocks. Since carbon stock changes in forests are disregarded here, the findings are only correct under the assumption that increasing wood construction does not have an effect on domestic wood removals from forests. If a growing inland market for construction wood actually led to additional fellings, GHG savings from an expanding building carbon stock would be diminished or entirely offset by forest carbon stock changes. However, since Austria's current net exports of sawnwood and panels are much higher than the maximum additional timber demand according to the presented scenarios, it is considered legitimate to assume a negligible effect on wood removals and equal forest carbon stock developments in all wood construction scenarios.

The possibility to recycle demolition wood for material purposes (‘cascading use’; [cf. 46) or to use it for energy generation is intentionally disregarded here. Since GHG savings compared to the ‘baseline’ scenario are investigated, and due to the long lifetimes of RB, the related GHG effects are negligible in the time frame until 2050 (see Figure 4). Nevertheless, it needs to be emphasized that especially cascading wood use provides considerable long-term GHG saving potentials [47–49].

### 5.3 Wood construction as climate mitigation measure

Werner *et al*. [23] argued that the carbon stock effect is of minor importance in comparison to substitution effects. This is correct under the following conditions: a short-lasting increase in wood utilization, rather than sustained growth; and if substitution factors do not decline considerably. However, for reasons explained above, life-cycle emissions of *all* building types will likely decrease in the future, resulting in declining substitution effects. Moreover, the trend toward wood construction in Austria has so far been a rather slow yet continuous process. If this trend is maintained until 2050, carbon stock effects in RB will in the end clearly dominate over substitution effects. To conclude, the question whether carbon stock or substitution effects are more relevant cannot be answered in general but has to be investigated for each case/scenario/application individually.

Considering the magnitude of Austria's total GHG emissions of about 80 Mt/a, it could be concluded from the above results that the effectiveness of wood construction as a climate mitigation measure is rather moderate. But it must be considered that the calculations are limited to building shells of residential houses. The GHG savings potential in non-residential building construction, which remains to be investigated, could be in a similar range. And increased wood use for interior works, furniture, doors and countless other long-lived products could further expand anthropogenic carbon stocks and reduce society's carbon footprint. The results show that promoting wood construction must be seen as a *long-term* mitigation measure. Its effectiveness in the short term is limited, but it could provide GHG savings when other (short-term) options are exhausted [cf. 50]. Furthermore, wood construction provides a valuable opportunity to offset GHG emissions from other activities. The feasibility of long-term decarbonization goals might depend on such options, as certain GHG sources exhibit high abatement costs. Therefore, promoting wood construction should be considered an integral part of a decarbonization strategy.

## Supplementary Material

Supplemental Material

## References

[1] FAO *Forestry for a Low-Carbon Future. Integrating Forests and Wood Products in Climate Change Strategies*. FAO Forestry Paper Food and Agriculture Organisation of the United Nations, Rome (2016).

[2] Sathre R, O'Connor J Meta-analysis of greenhouse gas displacement factors of wood product substitution. *Environ. Sci. Policy* 13(2), 104–114 (2010). doi:10.1016/j.envsci.2009.12.005.

[3] Werner F, Richter K Wooden building products in comparative LCA. A literature review. *Int. J. Life Cycle Assess*. 12(7), 470–479 (2007).

[4] Bergman R, Puettmann M, Taylor A et al The carbon impacts of wood products. *Forest Prod. J.* 64(7–8), 220–231 (2014). doi:10.13073/FPJ-D-14-00047.

[5] Hafner A, Schäfer S Comparative LCA study of different timber and mineral buildings and calculation method for substitution factors on building level. *J. Clean. Prod.* 167, 630–642 (2017). doi:10.1016/j.jclepro.2017.08.203

[6] Marcea RL, Lau KK Carbon dioxide implications of building materials. *J. Forest Eng.* 3(2), 37–43 (1992).

[7] Petersen AK, Solberg B Greenhouse gas emissions, life-cycle inventory and cost-efficiency of using laminated wood instead of steel construction.: Case: Beams at Gardermoen airport. *Environ. Sci. Policy* 5(2), 169–182 (2002).

[8] PPE (Projektplattform Energie) *Leitfaden 01: Ökologische Kenndaten Baustoffe Und Bauteile*. Projektplattform Energie des Bayerischen Bauindustrieverbandes e.V., Zentrum für Nachhaltiges Bauen (TU München), München (2015).

[9] Upton B, Miner R, Spinney M et al The greenhouse gas and energy impacts of using wood instead of alternatives in residential construction in the United States. *Biomass Bioenerg.* 32(1), 1–10 (2008). doi:10.1016/j.biombioe.2007.07.001.

[10] Suter F, Steubing B, Hellweg S Life cycle impacts and benefits of wood along the value chain: The case of Switzerland. *J. Ind. Ecol.* 21, 874–886 (2016). doi:10.1111/jiec.12486 PMC13061803 41971972

[11] Braun M, Fritz D, Weiss P et al A holistic assessment of greenhouse gas dynamics from forests to the effects of wood products use in Austria. *Carbon Manage.* 7(5–6), 271–283 (2016). doi:10.1080/17583004.2016.1230990

[12] ISO (International Organization for Standardization) *Greenhouse gases – Carbon footprint of products – Requirements and guidelines for quantification and communication. ISO/TS 14067:2013*. ISO, Geneva (2013).

[13] Kalt G, Höher M, Lauk C et al Carbon accounting of material substitution with biomass: Case studies for Austria investigated with IPCC default and alternative approaches. *Environ. Sci. Policy* 64(October), 155–163 (2016). doi:10.1016/j.envsci.2016.06.022.

[14] Skog KE, McKinley DC, Birdsey RA et al *Managing carbon*. USDA Forest Service/UNL Faculty Publications, Lincoln, Nebraska (2014).

[15] Hafner A, Rüter S, Schäfer S et al *Treibhausgasbilanzierung von Holzgebäuden – Umsetzung neuer Anforderungen an Ökobilanzen und Ermittlung empirischer Substitutionsfaktoren (THG-Holzbau). 148 S*. 28W-B-3-054-01 Waldklimafonds, Forschungsprojekt (2017). BMEL/BMUB. ISBN:978-3-00-055101-7

[16] Kalcher J, Praxmarer G, Teischinger A Quantification of future availabilities of recovered wood from Austrian residential buildings. *Resour. Conserv. Recycl.* 123, 143–152 (2016). doi:10.1016/j.resconrec.2016.09.001.

[17] Müller DB Stock dynamics for forecasting material flows—case study for housing in The Netherlands. *Ecol. Econ.* 59(1), 142–156 (2006). doi:10.1016/j.ecolecon.2005.09.025.

[18] Teischinger A, Stingl R, Berger V et al *Holzbauanteil in Österreich? Erhebung des Holzbauanteils aller Österreichischen Bauvorhaben* [Wood construction share in Austria? Survey investigating all construction projects in Austria] Institut für Holztechnologie und Nachwachsende Rohstoffe, Universität für Bodenkultur Wien, Vienna (2015).

[19] Rogelj J, den Elzen M, Höhne N et al Paris agreement climate proposals need a boost to keep warming well below 2°C. *Nature* 534(7609), 631–639 (2016). doi:10.1038/nature18307. 27357792

[20] Schleussner C-F, Rogelj J, Schaeffer M et al Science and policy characteristics of the Paris agreement temperature goal. *Nat. Clim. Change* 6(9), 827–835 (2016). doi:10.1038/nclimate3096.

[21] EC *Communication from the Commission to the European Parliament, the Council, the European Economic and Social Committee and the Committee of the Regions. Innovating for Sustainable Growth: A Bioeconomy for Europe. COM(2012) 60 Final*. European Commission, Brussels (2012).

[22] EC *Closing the Loop - An EU Action Plan for the Circular Economy. Communication from the Commission to the European Parliament, the Council, the European Economic and Social Committee and the Committee of the Regions. COM(2015) 614 final*. European Commission, Brussels (2015).

[23] Werner F, Taverna R, Hofer P et al Greenhouse gas dynamics of an increased use of wood in buildings in Switzerland. *Climatic Change* 74(1–3), 319–347 (2006). doi:10.1007/s10584-006-0427-2.

[24] German Environment Agency Submission under the United Nations Framework Convention on Climate Change and the Kyoto Protocol 2017. National Inventory Report for the German Greenhouse Gas Inventory 1990 – 2015. Climate Change 14/2017, Dessau-Roßlau (2017).

[25] Haberl H, Wiedenhofer D, Erb K-H et al The material stock–flow–service nexus: A new approach for tackling the decoupling conundrum. *Sustainability* 9(7), 1049 (2017). doi:10.3390/su9071049

[26] Krausmann F, Wiedenhofer D, Lauk C et al Global socioeconomic material stocks rise 23-fold over the 20th century and require half of annual resource use. *Proc. Natl. Acad. Sci*. 114(8), 1880–1885 (2017). 28167761 10.1073/pnas.1613773114 PMC5338421

[27] Müller A, Fritz S, Kranzl L *Energieszenarien bis 2050: Wärmebedarf der Kleinverbraucher. Ein Projekt im Rahmen der energiewirtschaftlichen Szenarien für den klima- und energiepolitischen Rahmen 2030 und 2050 und den Monitoring Mechanism 2017*. Energy Economics Group, Vienna University of Technology, e-think energy research, Vienna (2017).

[28] EC *Communication from the Commission to the European Parliament, the Council, the European Economic and Social Committee and the Committee of the Regions. Energy Roadmap 2050. COM(2011) 885/2*. European Commission, Brussels (2011a).

[29] EC *Communication from the Commission to the European Parliament, the Council, the European Economic and Social Committee and the Committee of the Regions; A Roadmap for Moving to a Competitive Low Carbon Economy in 2050. COM (2011) 112*. Brussels (2011b).

[30] UNFCCC (United Nations Framework Convention on Climate Change) *Adoption of the Paris Agreement, 21st Conference of the Parties*. United Nations / Framework Convention on Climate Change, Paris (2016).

[31] Arvesen A, Luderer G, Pehl M et al Deriving life cycle assessment coefficients for application in integrated assessment modelling. *Environ. Model. Software* 99, 111–125 (2018). doi:10.1016/j.envsoft.2017.09.010.

[32] Pehl M, Arvesen A, Humpenöder F et al Understanding future emissions from low-carbon power systems by integration of life-cycle assessment and integrated energy modelling. *Nat. Energy* 2(12), 939–945 (2017). doi:10.1038/s41560-017-0032-9.

[33] Müller A Energy demand assessment for space conditioning and domestic hot water: a case study for the Austrian building stock [PhD diss.]. Vienna University of Technology (2015).

[34] Passer A, Kreiner H, Maydl P Assessment of the environmental performance of buildings: A critical evaluation of the influence of technical building equipment on residential buildings. *Int. J. Life Cycle Assess*. 17, 1116–1130 (2012). doi:10.1007/s11367-012-0435-6.

[35] Jasinevičius G, Lindner M, Pingoud K et al Review of models for carbon accounting in harvested wood products. *Int. Wood Prod. J.* 6(4), 198–212 (2015).

[36] Pingoud K, Skog KE, Martino DL et al. *2006 IPCC Guidelines for National Greenhouse Gas Inventories. Volume 4: Agriculture, Forestry and Other Land Use, Chapter 12: Harvested Wood Products*. Intergovernmental Panel on Climate Change, Published: IGES, Japan (2006).

[37] Umweltbundesamt *Austria's National Inventory Report 2016. Submission under the United Nations Framework Convention on Climate Change and under the Kyoto Protocol*. Umweltbundesamt, Vienna (2016).

[38] Grêt-Regamey A, Hendrick E, Hetsch S et al *Challenges and Opportunities of Accounting for Harvested Wood Products. Background Paper to the Workshop on Harvested Wood Products in the Context of Climate Change Policies* jointly organized by the Swiss Federal Office for the Environment (FOEN), UNECE/FAO and MCPFE, Geneva, Switzerland (2008).

[39] BFW (Bundesforschungs- und Ausbildungszentrum für Wald, Naturgefahren und Landschaft) *Waldinventur 2007/09* [Forest inventory 2007/2009] BFW Praxis Information Nr. 24 - 2011 Vienna (2011).

[40] Strimitzer L, Höher M, Nemestothy K *Holzströme in Österreich 2015*. Austrian Energy Agency, Landwirtschaftskammer Österreich, Vienna (2017).

[41] WKÖ (Wirtschaftskammer Österreich) *Branchenbericht. Auf Holz klopfen – Solides Wachstum mit guten Perspektiven für das Jahr 2017* [Report of the Austrian wood industry 2017] Fachverband der Holzindustrie Österreichs, Vienna (2017).

[42] Landel P Modern timber construction in sweden. *Copenhagen*, 20 May (2015).

[43] Karjalainen M *Status and Possibilities of Wood Construction in Finland*. Ministry of Employment and the Economy, Strategic Programme for the Forest Sector, Helsinki (2015).

[44] Kohlmaier G, Kohlmaier L, Fries E et al Application of the stock change and the production approach to harvested wood products in the EU-15 countries: A comparative analysis. *European J. Forest Res.* 126(2), 209–223 (2007). doi:10.1007/s10342-006-0130-x.

[45] Lim B, Brown S, Schlamadinger B Carbon accounting for forest harvesting and wood products: Review and evaluation of different approaches. *Environ. Sci. Policy* 2(2), 207–216 (1999). doi:10.1016/S1462-9011(99)00031-3.

[46] Keegan D, Kretschmer B, Elbersen B, Panoutsou C Cascading use: a systematic approach to biomass beyond the energy sector. Biofuels, Bioproducts and Biorefining. 7(2), 193–206 (2013).

[47] Bais-Moleman AL, Sikkema R, Vis M et al Assessing wood use efficiency and greenhouse gas emissions of wood product cascading in the European union. *J. Clean. Prod.* 172, 3942–3954 (2018). doi:10.1016/j.jclepro.2017.04.153.

[48] Sathre R, Gustavsson L Energy and carbon balances of wood cascade chains. *Resour. Conserv. Recycl.* 47(4), 332–355 (2006).

[49] Sikkema R, Junginger M, McFarlane P et al The GHG contribution of the cascaded use of harvested wood products in comparison with the use of wood for energy—a case study on available forest resources in Canada. *Environ. Sci. Policy* 31(August), 96–108. doi:10.1016/j.envsci.2013.03.007.

[50] Kalt G, Baumann M, Lauk C et al Transformation Scenarios towards a Low-Carbon Bioeconomy in Austria. *Energy Strategy Rev.* 13–14(November), 125–133 (2016). doi:10.1016/j.esr.2016.09.004.

