# Supplementary material for: Carbon dynamics and GHG implications of increasing wood construction: long-term scenarios for residential buildings in Austria
Source: Carbon Manag. 2018 May 29;9(3):265–75. doi: 10.1080/17583004.2018.1469948 (PMC6397628; doi:10.1080/17583004.2018.1469948)
Supplement: Supplemental Material [file TCMT_A_1469948_SM2169.pdf]

Table A1. Model parameters

	Unit	Year		Sources and comments
		2015	2050	
**Population**	10^6	8.630	9.771	Statistik Austria (2017a)
**Floor space scenarios**	m^2 net floor space per person[2]			
'Trend'		44.6	47.7	Assumption based on A. Müller et al. (2017) and Kalcher et al. (2016) (scenario 'moderate increase');
'Decline'			42.0	Adopted from Kalcher et al. (2016) (scenario 'moderate decline');
**Wood construction scenarios**				Wood construction shares in 2010 and 2015 are inter-/extrapolated values based on Teischinger et al. (2015); shares after 2015 are scenario assumptions. Timber intensities are based on wood construction shares and specific timber demand for typical buildings derived from the construction element database 'Baubook' (2017). The assumed average timber intensities of single-/two- and multi family-buildings are 0.23 m^3/m^2 gross floor space (single-/two-family) and 0.14 m^3/m^2 (multi-familiy) for wood construction, and 0.04 m^3/m^2 and 0.02 m^3/m^2 for conventional construction. The resulting average timber intensity over all RB in the base year 2015 is in good agreement with the average value for the timeframe 2001 to 2010 derived from Kalcher et al. (2016): 0.10 m^3/m^2.
Scenario 'Baseline'				
Wood construction share	%	22.0%	22.0%	
Avarage timber intensity[1]	m^3/m^2	0.108	0.108	
Scenario 'Continued increase'				
Wood construction share	%	22.0%	50.0%	
Avarage timber intensity[1]	m^3/m^2	0.108	0.180	
Scenario 'Rapid increase'				
Wood construction share	%	22.0%	80.0%	
Avarage timber intensity[1]	m^3/m^2	0.108	0.257	
**Substitution factor scenarios**	GHG savings in kg CO_2-equ. per m^2 net floor space			
'Default & dynamic'		142.9	28.6	Standard assumption derived from Hafner et al. (2017)
'High & dynamic'		200.0	40.0	Alternative assumptions (based on ranges according to Hafner et al. [2017]) assumed for sensitivity analyses. Constant substitution factors are considered highly unlikely (grey font color).
'Low & dynamic'		93.3	18.7	
'Default & constant'		142.9	142.9	
'High & constant'		200.0	200.0	
'Low & constant'		93.3	93.3	

1) Timber intensity is measured in m^3 of timber per m^2 net floor space. Data refer to the buildings constructed in the respective year, not the entire building stock.

2) Default ratio net to gross floor space: 0.7; Assumptions for sensitivity analyses regarding substition factors: 0.65 to 0.75

Table A2. Cumulated GHG savings during 2015 to 2050 in all scenarios and sensitivity analyses (Unit: Mt CO_2-equ.). The most relevant results, which are also referred to in the abstract, are highlighted in yellow.

Wood construction scenario	Substitution factor scenario	Living space scenario	Cumulated GHG savings from material substitution effect during 2015 to 2050: Savings	Cumulated GHG savings from material substitution effect during 2015 to 2050: Difference to respective 'Baseline' scenario	Total cumulated GHG savings 2015 to 2050: Savings	Total cumulated GHG savings 2015 to 2050: Difference to respective 'Baseline' scenario
**Main scenarios**						
'Baseline'	'Default & dynamic'	'Trend'	4.89	-	17.32	-
		Decline'	3.85	-	10.74	-
'Continued increase'	'Default & dynamic'	'Trend'	7.89	3.00	29.55	12.23
		Decline'	6.04	2.19	19.54	8.80
'Rapid increase'	'Default & dynamic'	'Trend'	10.96	6.07	42.22	24.90
		Decline'	8.27	4.42	28.63	17.89
**Sensitivity analyses**						
'Baseline'	'Low & dynamic'	'Trend'	3.19	-	15.63	-
		Decline'	2.52	-	9.41	-
	'High & dynamic'	'Trend'	6.84	-	19.28	-
		Decline'	5.39	-	12.28	-
	'Default & constant'	'Trend'	8.05	-	20.49	-
		Decline'	7.38	-	14.27	-
	'Low & constant'	'Trend'	5.26	-	17.70	-
		Decline'	3.99	-	10.88	-
	'High & constant'	'Trend'	11.27	-	23.71	-
		Decline'	8.54	-	15.44	-
'Continued increase'	'Low & dynamic'	'Trend'	5.15	1.96	26.82	11.19
		Decline'	3.94	1.43	17.45	8.04
	'High & dynamic'	'Trend'	11.04	4.20	32.71	13.43
		Decline'	8.45	3.06	21.95	9.67
	'Default & constant'	'Trend'	14.24	6.18	35.91	15.42
		Decline'	12.92	5.54	26.42	12.15
	'Low & constant'	'Trend'	9.30	4.04	30.97	13.27
		Decline'	6.88	2.89	20.38	9.50
	'High & constant'	'Trend'	19.93	8.66	41.60	17.89
		Decline'	14.74	6.19	28.24	12.80
'Rapid increase'	'Low & dynamic'	'Trend'	7.16	3.97	38.42	22.79
		Decline'	5.40	2.89	25.76	16.35
	'High & dynamic'	'Trend'	15.34	8.50	46.60	27.33
		Decline'	11.58	6.19	31.94	19.65
	'Default & constant'	'Trend'	20.66	12.61	51.92	31.43
		Decline'	18.66	11.28	39.02	24.75
	'Low & constant'	'Trend'	13.50	8.24	44.76	27.06
		Decline'	9.87	5.88	30.23	19.35
	'High & constant'	'Trend'	28.93	17.65	60.19	36.48
		Decline'	21.15	12.61	41.51	26.07
